# No effect of running and laboratory housing on adult hippocampal neurogenesis in wild caught long-tailed wood mouse

**DOI:** 10.1186/1471-2202-10-43

**Published:** 2009-05-06

**Authors:** Thomas Hauser, Fabienne Klaus, Hans-Peter Lipp, Irmgard Amrein

**Affiliations:** 1 Institute of Anatomy, University of Zürich, Winterthurerstrasse 190, 8057 Zürich, Switzerland

## Abstract

**Background:**

Studies of adult hippocampal neurogenesis (AHN) in laboratory rodents have raised hopes for therapeutic interventions in neurodegenerative diseases and mood disorders, as AHN can be modulated by physical exercise, stress and environmental changes in these animals. Since it is not known whether cell proliferation and neurogenesis in wild living mice can be experimentally changed, this study investigates the responsiveness of AHN to voluntary running and to environmental change in wild caught long-tailed wood mice (*Apodemus sylvaticus*).

**Results:**

Statistical analyses show that running had no impact on cell proliferation (p = 0.44), neurogenesis (p = 0.94) or survival of newly born neurons (p = 0.58). Likewise, housing in the laboratory has no effect on AHN. In addition, interindividual differences in the level of neurogenesis are not related to interindividual differences of running wheel performance (r_s _= -0.09, p = 0.79). There is a correlation between the number of proliferating cells and the number of cells of neuronal lineage (r_s _= 0.63, p < 0.001) and the number of pyknotic cells (r_s _= 0.5, p = 0.009), respectively.

**Conclusion:**

Plasticity of adult neurogenesis is an established feature in strains of house mice and brown rats. Here, we demonstrate that voluntary running and environmental changes which are effective in house mice and brown rats cannot influence AHN in long-tailed wood mice. This indicates that in wild long-tailed wood mice different regulatory mechanisms act on cell proliferation and neurogenesis. If this difference reflects a species-specific adaptation or a broader adaptive strategy to a natural vs. domestic environment is unknown.

## Background

In the dentate gyrus of the mammalian hippocampus progenitor cells continuously generate neurons throughout adulthood [1]. Adult hippocampal neurogenesis (AHN) occurs in various investigated mammals [2], including primates [3] and humans [4] but is low or missing in bats [5]. Among wild living mice and voles, levels of AHN can vary to a great extent, but it remains similar in taxonomic closely related species [6,7]. Two main findings have emerged from studies in laboratory mice and rats on the function of these new neurons and the regulation of AHN. First, the functional role of newly generated neurons has remained controversial. There have been reports that AHN correlates positively with hippocampus-dependent learning tasks, especially with spatial learning [8-11]. On the other hand, complete elimination of adult hippocampal neurogenesis has no or only minimal effects on a large number of behavioural measures in mice [12]. Second, adult neurogenesis in laboratory rodents varies extensively between species and strains [13,14], and can be modulated by various internal and external factors. Triggers such as physical exercise, enriched environment [15,16] and growth factors like BDNF or IGF-1 stimulate AHN [17]. Age, stress and impoverished environment in contrast have a negative regulatory effect on the production of new neurons [18-20].

Physical exercise is a strong stimulator of cell proliferation and neurogenesis. In laboratory rodents it enhances the amount of cell proliferation and neurogenesis up to three fold [21]. These rodent findings have attracted much interest, because triggering of AHN through physical exercise might improve cognitive abilities in disabled or healthy humans, possibly by inducing specific gene expression patterns [22,23].

However, it remains an open question whether experimental data from rodents can be extrapolated to humans, or which species could serve as animal model -laboratory or wild rodents. It is also not known whether cell proliferation and neurogenesis in wild living mice can be experimentally modulated. Thus, this study investigates the responsiveness of AHN to voluntary running in the wild caught long-tailed wood mouse (*Apodemus sylvaticus*). Furthermore, we investigated whether individual differences in neurogenesis and level of voluntary exercise are related, and if environmental changes alter AHN.

The long tailed wood mouse is genetically one of the closest relative to *Mus musculus *[24]. Ecologically, it is also one of the best-studied wild mouse species. Wood mice are commonly distributed throughout Europe, parts of Asia and north-western Africa. They are characterized as agile animals with patrolling behaviour and good spatial memory having a territory size up to 25'000 m^2 ^[25,26]. In behavioural tests wood mice show moderately better learning abilities compared to bank voles [27].

In this study, proliferating cells were visualized with the endogenous marker Ki-67 [28-30], a chromosome-associated protein present during the active phase of the cell cycle (G_1_-M). Developing neurons were immunohistochemically detected with a marker against doublecortin (DCX). DCX is a microtubule-associated protein expressed in migrating cells and during the initial period of morphological maturation and functional integration of the developing neuron [31-33]. It has been shown that DCX is a reliable marker for neurogenesis since it monitors specifically progenitor cells of neuronal lineage and young neurons [34]. Pyknotic cells were visualized using a Giemsa stain.

We compared the relation of wheel running activity during 14 days and AHN in wild caught wood mice. In order to test for effects of captivity, AHN was assessed in animals immediately after capturing and compared to AHN in animals kept in home cages with and without running wheels.

## Results

### Exercise and cell proliferation, neurogenesis and apoptosis

**Figure 1:**
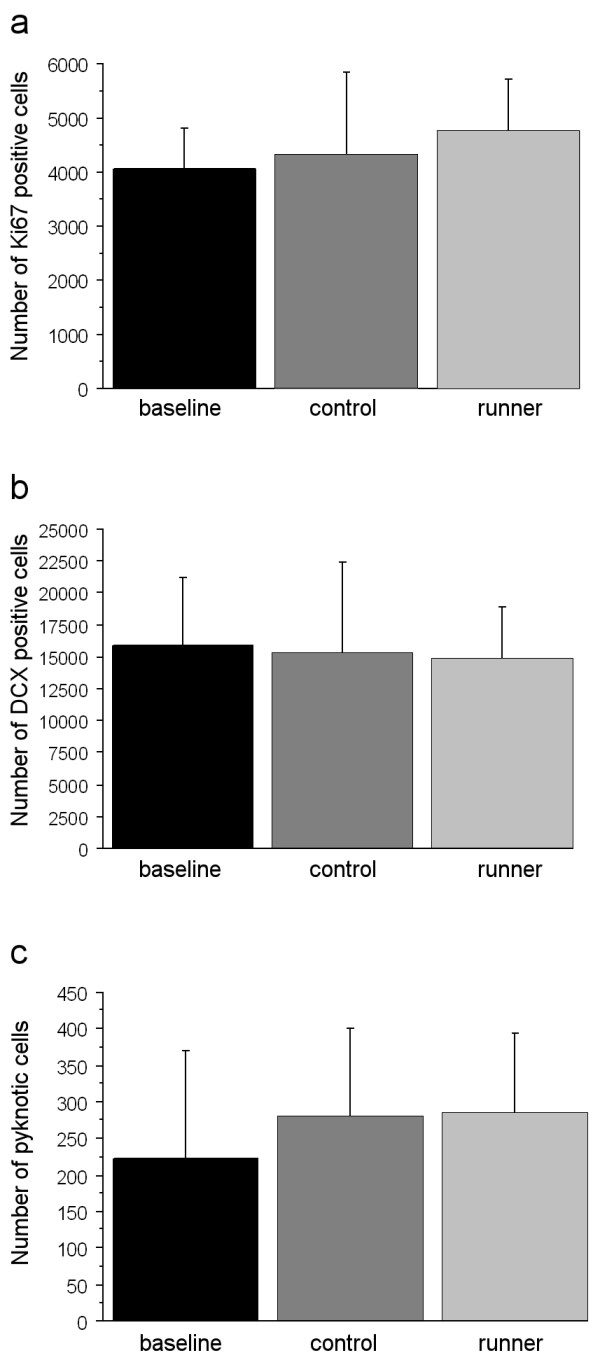
**Group comparison of cell numbers**. Experimental groups (baseline = investigated directly after trapping; control = two weeks of laboratory housing; runner = two weeks of voluntary running) do not differ in their number of proliferating cells (a, Ki67), young neurons (b, DCX) and pyknotic cells (c). Analysis was performed with general linear model. Bars represent SD.

**Figure 2:**
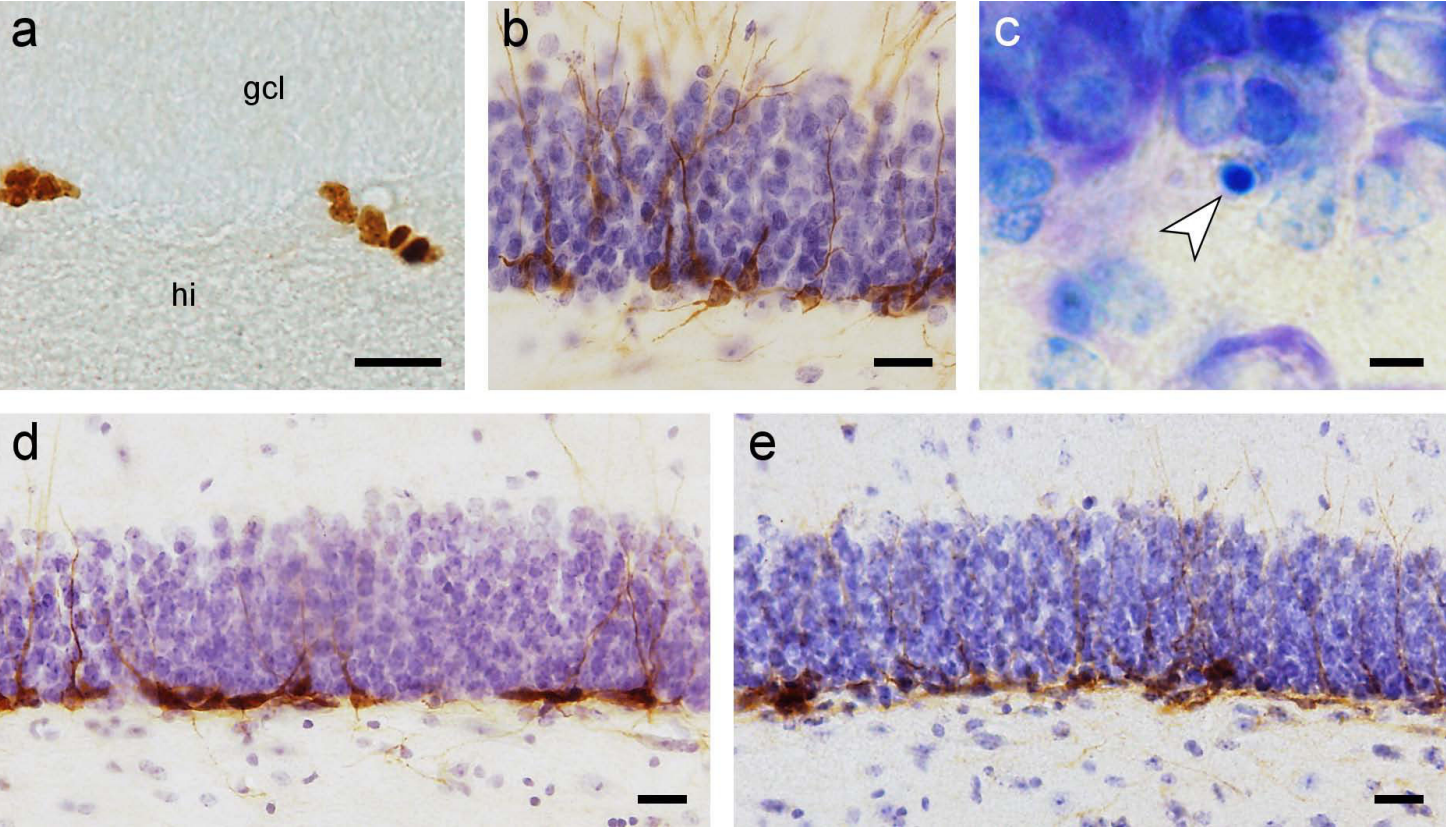
**Immunohistochemistry staining**. **a**: Ki67 staining of the septal part of the dentate gyrus subgranular zone. Two clusters of proliferating cells, indicated in brown, are located in the subgranular zone (gcl: granule cell layer; hi: hilus). Scale bar = 20 μm. **b**: DCX positive cells (brown) in the septal part of the dentate gyrus subgranular zone. The DCX immunostaining is counterstained with haematoxylin. Young neurons extend long dendrites into the granular and molecular cell layer. Scale bar = 20 μm **c**: Condensed chromatin of a pyknotic cell in the subgranular zone is visualized with Giemsa staining in plastic embedded tissue. Arrow indicates a pyknotic cell. Scale bar = 5 μm **d**: Qualitative comparison of the amount of young neurons (stained in brown) in the subgranular zone of a baseline wood mouse (**d**) and a running wood mouse (**e**). No difference in the number of DCX positive cells can be seen. Scale bar = 20 μm.

The three experimental groups, baseline, control and running group, (see Methods) were tested for differences in cell numbers. Statistic analysis of Ki67 positive cells (Fig. 1a) showed no difference in proliferation rates between the three experimental groups (p = 0.443). Analysis of neurogenesis, using DCX as a marker for cells of neuronal lineage (Fig. 1b), also showed no group differences (p = 0.938). Likewise, comparison of the number of pyknotic cells (Fig. 1c) revealed no significant differences (p = 0.576). Data of cell counts are shown in Table 1, visualization of immunohistochemical stains are shown in Fig. 2 for Ki67 (Fig. 2a), DCX (Fig. 2b, d, e), and a Giemsa-stained pyknotic cell (Fig. 2c).

**Table 1:** Estimates of proliferating cells, young neurons and pyknotic cells in the dentate gyrus

	**baseline**	**control**	**running**	CE	Sampling sites*	Sections analyzed*	Cells counted*
**Ki67**	4'047	4'315	4'744	0.049		14 (12–15)	695 (356–1001)
SD	772	1'525	977				
**DCX**	15'840	15'204	14'871	0.09	339(273–486)	14 (10–16)	157 (64–319)
SD	5'426	7'238	4'019				
**pyknotic**	223	279	283	0.09		27 (21–30)	48 (17–78)
SD	148	122	110				

Average numbers of young neurons, visualized immunohistochemically through Doublecortin (DCX), are obtained from stereological estimates using the optical fractionator method. Proliferating cells (Ki67 positive cells) and pyknotic cells are counted manually and multiplied by the section fraction to estimate total cell numbers. All parameters were analyzed in every sixth section. SD stands for standard deviation and CE is coefficient of error. *Values are means with ranges in parentheses.

### Correlation of the numbers of proliferating cells, cells of neuronal lineage and pyknotic cells

Analysis of the pooled data from all experimental groups showed a strong correlation between the number of proliferating cells and the number of cells of neuronal lineage (r_s _= 0.63, p < 0.001). Group-separated analysis revealed a correlation in both the running (r_s _= 0.66, p = 0.026) and the control group (r_s _= 0.78, p = 0.013). Correlation in the baseline group was marginally significant (r_s _= 0.75, p = 0.052). Total numbers of pyknotic cells and proliferating cells were also correlated (r_s _= 0.50, p = 0.009), but there was no correlation between the number of DCX positive- and pyknotic cells (r_s _= 0.18, p = 0.387).

### Running and cell parameters

**Figure 3:**
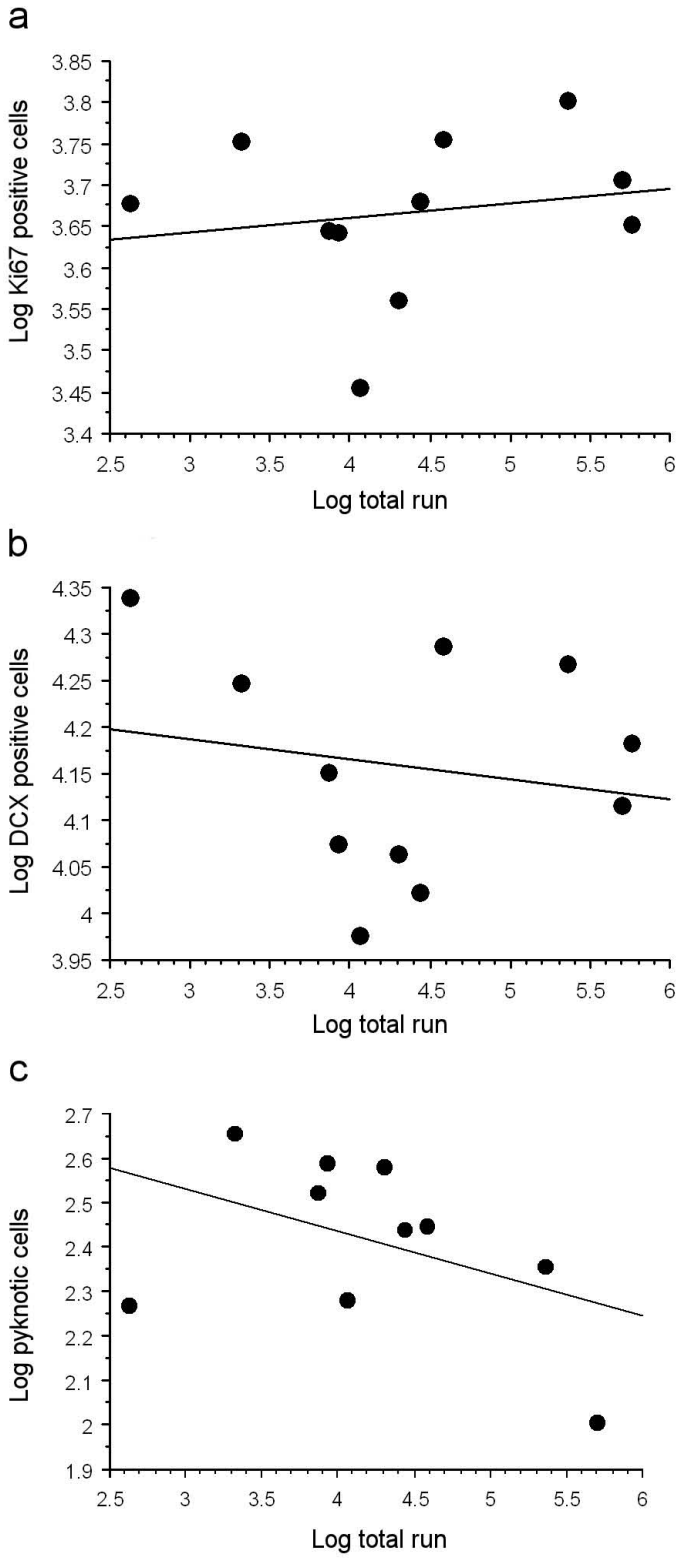
**Performance and cell parameters**. There is no correlation between performance (log total run) over the two weeks and the amount of proliferating cells (a, Ki67 positive cells), young neurons (b, DCX positive cells) and pyknotic cells (c). Data are represented in logarithmic numbers.

There was no correlation between performance and the amount of Ki-67 positive cells (r_s _= 0.29, p = 0.39, Fig. 3a), cells of neuronal lineage (r_s _= -0.09, p = 0.79, Fig. 3b) and pyknotic cells (r_s _= -0.38, p = 0.28, Fig. 3c), respectively. Performing mice could be grouped in three activity classes. Five mice were allocated to the first category that did less than an average of 1000 revolutions per day. The second category comprised three animals that ran between 1000–3000 revolutions, whereas the third category included three animals that performed more than a daily amount of 10'000 revolutions on average. Daily running activity of each individual varied considerably. On average, the wild animals performed significantly less than C57BL/6 laboratory mice, under identical experimental conditions, (p = 0.047; Klaus et al., in preparation).

### No gender specific difference within experimental groups

Within the limits of statistical power set by the low number of females in this study, no gender dependent difference were found in any experimental group for any of the parameters investigated (data not shown).

## Discussion

In this study we investigated for the first time whether wheel running has an impact on cell proliferation, neurogenesis and cell death in wild long-tailed wood mice, but found none.

### Voluntary running affects cell proliferation and neurogenesis in laboratory rodents but not in wood mice

Physical exercise leads to neuromorphological and neurobehavioural alterations in the brain of laboratory rodents. It increases cell proliferation and neurogenesis [16,35], enhances dendritic spine density on granule cells [36,37], stimulates neurotrophic factor activity [22,38] and correlates with improved cognitive functions in spatial [35,39,40] and non-spatial memory tasks [41,42]. In rats, hippocampal BDNF is increased after two nights of free access to a running wheel [23] and a single week of exercise is sufficient to improve memory performance in the water maze task [43]. The effects of physical exercise on cell proliferation and neurogenesis, as well as neurobehavioral features, are age-independent [41,44-46].

Here, we demonstrate that in wild wood mice voluntary running has no impact on cell proliferation, neurogenesis and apoptosis in the hippocampus. Not only does physical exercise not influence cell proliferation and neurogenesis, but AHN seems also resistant to changes in the environment as indicated by the lack of differences between animals sacrificed immediately after trapping and those housed in the laboratory.

### Why is there no exercise effect in wood mice?

Exercise-induced increase in neurogenesis and cell proliferation may be counterbalanced by stress, which has a suppressive effect on granule cell genesis [47]. However, as the extreme environmental change from natural living conditions to laboratory housing, be it with or without running wheel, did not cause changes in any AHN-related cellular processes, we expect that stress is not involved. In daily home cage observation we did not see any sign of stress or discomfort. No stereotypic behaviour could be detected [48]. In addition a previous study with wild wood mice showed that short-term captivity is not stressful enough to decrease adult neurogenesis [7].

In the natural habitat of wild wood mice daily running belongs to their behavioural pattern [25]. If running stimulates cell proliferation and neurogenesis similarly in wild mice as it does in laboratory rodents it might be argued that proliferation and neurogenesis in the natural habitat proceed at a level that, under laboratory conditions, is just being maintained by access to a running wheel. Therefore, a ceiling effect of AHN, reached through natural activity, might be the reason why running under laboratory conditions does not result in a further enhancement, as the production of new neurons would be already at the maximum. However, individual performance of wood mice varied considerably without effecting AHN. Some mice ran on a low level of about 100–3000 revolutions while other animals performed on higher levels of 16'000–40'000 revolutions on average. In laboratory mice, three hours of performance (~3500 mean daily wheel revolutions) is enough to significantly increase cell proliferation, cell survival and total number of new neurons [44]. Restricting the analysis to wild mice which ran between 3500 and 63'000 revolutions per active phase, no exercise related alteration was found in these animals when compared to non runners or mice of the baseline group. Even if low performance might be enough to maintain a natural high level of AHN in wild mice, we would expect a decrease of AHN in animals kept without access to a running wheel, which is not the case. The low rho values of all correlations also stress the independence of cell proliferation, neurogenesis or cell death from physical activity.

Natural living conditions may regulate AHN over a time span that outlast the experimental period of two weeks. A long-term regulation that maintains a constant pool of functionally distinct young neurons may be an adaptive strategy in the face of constantly changing natural living conditions. To our knowledge there is no data on the duration of a running-induced effect on adult neurogenesis in house mice and brown rats once the exercise has ceased. In this context it is remarkable that laboratory housing of wild wood mice does not lead to alterations in AHN despite the environmental change from nature to laboratory cage, which one could consider as a severe loss of environmental stimuli.

### AHN stimulation by running – a species-specific effect or a trait of domestication?

In laboratory mice regulatory mechanisms of proliferation and neurogenesis are related to the genetic background [13,14], which in turn is the result of unintentional selective breeding that can have profound consequences on physiology, morphology and behaviour. It is unknown if the adaptive response of AHN to stimulating factors is a naturally occurring feature which laboratory animals have inherited from their ancestors. At least, the finding that running and environmental changes affect AHN in laboratory rodents but not in wild wood mice indicates that different regulatory mechanisms are operative in these species. Another possible source for regulation differences between wild and laboratory rodents is domestication. Domestication is accompanied by an irreversible reduction of 10.2% of hippocampus volume in rats [49] and changes in behaviour including a reduced sensitivity to predators in rodents, suggesting diminished anxiety and fear responses [50]. In a long-term study of selective breeding exclusively for tameness in silver foxes, Trut (1999) has shown that in the course of domestication the timing of the postnatal development of neurochemical and neurohormonal mechanisms changes [51]- alterations that have the potential to affect the development of the hippocampus as one of the brain structures that differentiate late in ontogenesis.

## Conclusion

Although this study is limited by the fact that we only investigated one species, it emphasizes the importance to examine AHN in a broader, evolutionary context [52]. Attempts to translate data from rodents to man in the field of adult neurogenesis should be based on a large variety of possible regulatory mechanisms. Studies in humans are limited for obvious reasons, and it is not clear if humans should be characterized as domesticated or wild, or if this characterization is required at all. Studying factors regulating AHN in different rodent species is a necessary step in charting white spots on the map of regulatory pathways of AHN. The finding that neither running nor environment influence adult hippocampal neurogenesis in wild long-tailed wood mice (one of the closest relatives of house mice) indicates that different regulatory mechanisms are operative in this species compared to laboratory mice and rats. Whether these findings reflect domestication effects, specific genetic background or species-specific environmental adaptation of the animals is yet unknown. More studies on various wild mouse species are required to verify differences in regulatory mechanisms of AHN between wild and laboratory mice, hopefully elucidating the source of these differences. The widespread use of laboratory rodents in translational research on AHN makes it important to address these questions.

## Methods

### Animal

27 wood mice (*Apodemus sylvaticus*), 21 males and six females, were trapped in live-traps in the park around the University of Zürich in spring. Traps were controlled every 2 hours and mice either immediately perfused **(baseline group**, total 7 (5 m/2 f)**) **or singly housed for 14 days in cages containing either a running wheel **(running group**, total 11 (9 m/2 f)**) **or no environmental enrichment **(control group**, total 9 (7 m/2 f)**)**. Mice had free access to running wheels (∅ 14 cm). All cages were provided with bedding material, fresh hay, water and standard laboratory mouse food pellets supplemented with fruits and seeds. Mice were exposed to a 12 hours light-dark phase. Wild animals were classified as juvenile, adult or old based on tooth wear, sexual maturity, marks of previous pregnancies and body weight in correlation to season as done and described before [6]. All experiments were approved by the veterinary office of the Kanton of Zürich.

### Histology

Mice of the running and control groups were sacrificed immediately after the active phase. They were anaesthetized with Pentobarbital (50 mg/kg body weight) and perfused transcardially with cold phosphate-buffered saline (PBS) followed by 0.6% sodium sulphide solution in PBS and cold 4% paraformaldehyde with 15% saturated picric acid in PBS. Brains were removed, hemispheres separated and postfixed over night. After postfixation, right hemispheres were transferred into 30% sucrose in PBS and frozen after saturation. For immunohistochemistry 40 μm sagittal sections were cut and stored at -20°C in a cryoprotective solution until further processing. For Giemsa staining left hemispheres were dehydrated for a total of 9 h in alcohol (4 × 70%, 4 × 96%, 2 × 99%), incubated for ten days in infiltration solution and embedded in glycomethacrylate (Technovit 7100, Heraeus Kulzer GmbH, Wehrheim, Germany).

#### Ki67 and DCX staining

For Ki67 immunohistochemistry, free floating sections were incubated for epitope retrieval in citrate buffer, pH 6.0, at 90°C for 40 min, followed by incubation in endogenous peroxidase blocking reagent, 0.6% H_2_O_2 _in TBS-Triton (0.05% Triton X-100 in TBS, pH 7.4) for 30 min at room temperature (RT). For DCX immunohistochemistry, free floating sections were microwaved at 600 W in citrate buffer, pH 6.0, for 1.5 min for epitope retrieval, followed by incubation in endogenous peroxidase blocking reagent (see above). Thereafter sections were preincubated in 2% Serum (for Ki67: NGS; for DCX: NRS) + 0.1% BSA + 0.25% Triton in TBS, for 60 min at RT. Afterwards, sections were incubated with primary antibody Ki67 (polyclonal rabbit NCL-Ki67p, Novocastra, 1:5000 in preincubation solution) and DCX (polyclonal goat IgG, Santa Cruz Biotechnology, 1:1000 in preincubation solution) overnight at 4°C. Incubation with secondary antibodies (for Ki67: biotinylated goat anti-rabbit IgG 1:1000 + 2%NGS + 0.1%BSA in TBS; For DCX: rabbit anti-goat IgG, Vectastain Elite ABS Kit, 1:1000 + 2%NRS + 0.1%BSA in TBS) was performed for two hours followed by incubation with streptavidin-biotin complex (Vectastain Elite ABC kit) and stained with DAB as chromogen. Until incubation with the primary antibody all rinses in between incubations were made with TBS-Triton, afterwards with TBS alone.

#### Giemsa staining

Glycomethacrylate-embedded left hemispheres were cut horizontally at 20 μm with a metal knife on a Leitz Rotary microtome. Every sixth section was stained according to the protocol of Iniguez [53]. Incubation in Giemsa staining solution (Giemsa stock solution 1.09204.0500, Merck, Darmstadt, Germany) diluted 1: 10 in buffer (67 mmol KH2PO4) at RT for 40 min., rinsed in 1% acetic acid for 10 sec. and differentiated in 3 × 99% alcohol, cleared in Xylol and mounted with Eukitt.

### Measurements

#### Run distance

A controller system (AMS Software and Electronic GmbH, Flensburg Germany) registered the animal's running activity in one hour bins.

#### Total DCX positive cell number

The number of DCX positive cells was estimated in every sixth section using the optical fractionator [54] with StereoInvestigator software (MicroBrightField Inc. Williston, USA). This stereological method provides unbiased estimates of neuron number. Assumptions about neuron size and shape are not necessary and estimates are unaffected by tissue shrinkage. The microscope used was a Zeiss Axioplan with a 100× oil-immersion lens. Cells were counted in a frame of 30 – 30 μm with a x, y-step size of 120 μm. Total DCX-positive cell number (N) is calculated using the formula N = ∑Q^- ^× (t/h) × (1/asf) × 1/ssf, where Q^- ^= total number of cells counted, t = section thickness, h = height of optical disector, asf = area of sampling fraction = a(frame)/a(x, y step) and ssf = section sampling fraction.

#### Total Ki67 positive cell number

Proliferating cells were counted exhaustingly in every sixth section on an Olympus light microscope using a 63× oil-immersion lens and multiplied by the section sampling fraction to obtain estimated total cell number. Cells in the top focal plane of the section were not counted. All Ki67 positive cells in the subgranular layer (SGL) and in the granule cell layer (GCL) (reaching 1/3 into cell layer; towards molecular layer) of the right hemisphere were counted.

#### Total pyknotic cell number

Estimates of total apoptotic cells were made as described for Ki67, using a Zeiss Axioplan microscope with a 40× oil-immersion lens. Pyknotic cells were easily identified by their strongly stained nuclei whose chromatin condensed into peripherally (C or doughnut shape), solid or multiple cell bodies [7]. Pyknotic cells were counted manually in the same zone as proliferating cells. Again, cells in the top focal plane of the section were not considered.

### Statistics

Only adult animals are included in statistical calculations. Group comparisons of total number of proliferating cells, new born neurons and pyknotic cells were performed with general linear model (GLM). Statistical significance level was determined at 5%. Correlation analyses were performed with paired two-group Spearman rank correlation. Statistical analyses were performed with SPSS software (version 17).

The brightness and contrast of microphotographs were adjusted to resemble the appearance of the sections under the microscope. No local changes were made to the images.

## Authors' contributions

TH and FK participated in the design of the study, trapped and perfused the mice, carried out the experiment, cut the brains and processed sections for immunohistochemistry. Furthermore, they performed statistical analysis, interpreted data and drafted manuscript. HPL provided the funding and participated in writing the manuscript. IA conceived of the study, helped designing it and participated in writing the manuscript. All authors read and approved the final manuscript.
